# Lack of EGF receptor contributes to drug sensitivity of human germline cells

**DOI:** 10.1038/sj.bjc.6602315

**Published:** 2005-01-11

**Authors:** S-J Park, S Armstrong, C-H Kim, M Yu, K Robertson, M R Kelley, S-H Lee

**Affiliations:** 1 Department of Biochemistry and Molecular Biology, Indiana University School of Medicine, Indianapolis, IN 46202, USA; 2 Walther Oncology Center, Indiana University School of Medicine, Indianapolis, IN 46202, USA; 3 Department of Medicine-Biostatistics, Indiana University School of Medicine, Indianapolis, IN 46202, USA; 4 Department of Pediatrics, Indiana University School of Medicine, Indianapolis, IN 46202, USA

**Keywords:** DNA damage, epidermal growth factor receptor, drug resistance, cisplatin, cancer, chemotherapy

## Abstract

Germline mutations have been associated with generation of various types of tumour. In this study, we investigated genetic alteration of germline tumours that affect the drug sensitivity of cells. Although all germline tumour cells we tested were hypersensitive to DNA-damaging drugs, no significant alteration was observed in their DNA repair activity or the expression of DNA repair proteins. In contrast, germline tumours expressed very low level of epidermal growth factor receptor (EGFR) compared to drug-resistant ovarian cancer cells. An immunohistochemical analysis indicated that most of the primary germline tumours we tested expressed very low level of EGFR. In accordance with this, overexpression of EGFR in germline tumour cells showed an increase in drug resistance, suggesting that a lack of EGFR, at least in part, contributes to the drug sensitivity of germline tumours.

Germline mutations are associated with generation of various tumours. Previous studies indicated that germline cells were hypersensitive to DNA-damaging agents (Cagnoli *et al*, 1998). Although the mechanisms, of drug resistance are still poorly understood, an increased rate of DNA adduct removal appears to be associated with drug resistance in various human cancers (Lai *et al*, 1988; Dinapoli *et al*, 1993; Johnson *et al*, 1994; Eastman and Schulte, 1998). Drug-resistant human tumours have been shown to express higher levels of nucleotide excision repair (NER) proteins such as XPA, XPB (Lai *et al*, 1988; Eastman and Schulte, 1998), ERCC1, and cockayne syndrome group B (CSB) (Lai *et al*, 1988). Also, altered expression of genes involved in *O*-6-alkyltransferase-mediated direct DNA repair (*O*-6-methylguanine DNA methyl transferase, MGMT) or base excision repair pathway also contributes to drug resistance of cancer cells (Chetsanga and Lindahl, 1979; Doetsch and Cunningham, 1990; Cohen *et al*, 1991; Demple and Harrison, 1994; Gill *et al*, 1996; Deutsch *et al*, 1997; Asagoshi *et al*, 2000; Bielas and Heddle, 2000; Dobson *et al*, 2000; Evans *et al*, 2000). Defects in mismatch repair (MMR) are associated with cisplatin resistance by contributing to increased replication bypass of cisplatin adducts and to a drug-tolerant phenotype (Hansen *et al*, 1998; Karahalil *et al*, 1998; Hansen and Kelley, 2000; Limp-Foster and Kelley, 2000; O'Neill, 2000). Therefore, loss of MMR proteins such as hMLH1 leads to resistance of tumour cells to a variety of DNA-damaging agents, including bifunctional alkylating and monofunctional methylating agents (Rosenquist *et al*, 1997; Waters *et al*, 1999; Zharkov *et al*, 2000) such as cisplatin and *N*′-methyl-*N*-nitrosourea (Koi *et al*, 1994; Fishel and Kolodner, 1995; Fink *et al*, 1996, 1998; Drummond *et al*, 1996).

In addition to genetic alteration of DNA repair genes, altered drug transport, increased metallothionein or glutathione levels, mitochondrial alterations, and altered DNA adduct formation have been reported to contribute to drug resistance of human cancers (Graves *et al*, 1992; Mello *et al*, 1996; Kuga *et al*, 1997; Leighton *et al*, 1997; Mitra *et al*, 1997; Umar *et al*, 1997). Growth factor receptor such as epidermal growth factor receptor (EGFR) is also amplified in many solid tumours (Arteaga, 2003). Introduction of a protein tyrosine kinase inhibitor selectively blocks proliferation of EGFR-expressing tumour cells, suggesting a role for EGFR in tumour cell growth (Lydon *et al*, 1998). Epidermal growth factor receptor is necessary for cisplatin-mediated apoptosis in tumour cells, suggesting a possible involvement of EGFR pathway in mediating the repair of drug-induced DNA damage(s) (Dixit *et al*, 1997). On the other hand, overexpression of EGFR family members suppressed the antiproliferative/cytotoxic activity of tumour necrosis factor (TNF)-alpha, suggesting that it may have an antagonistic role in TNF pathway (Perez and Donato, 1996; Hoffmann *et al*., 1998). Nonetheless, overexpression of EGFR observed in human breast/ovarian tumours is associated with poor prognosis with cancer patients (Baekelandt *et al*, 1999; Witters *et al*, 1999).

In this study, we investigated the genetic alteration of germline tumours such as altered DNA repair activity and/or damage signalling pathways that affect the drug sensitivity of cells. We found no significant change in NER activity or expression of DNA repair proteins in drug-sensitive germline cells compared to the drug-resistant ovarian cancer cells. Instead, the expression of a membrane receptor tyrosine kinase, EGFR, correlated with the cells drug resistance. Drug-resistant cancer cells exhibited elevated level of EGFR expression, while drug-sensitive germline cells showed a lower EGFR expression. Overexpression of the EGFR gene significantly enhanced the cells drug resistance, suggesting that EGFR may be one of the contributing factors that affect drug resistance of cancer cells.

## MATERIALS AND METHODS

*Cell lines, cell culture, and drug treatment*: NT2/D1 cells were obtained from American Type Culture Collection (Rockville, MD, USA) and 833K and 64CP9 GCT cell lines were obtained from G Sledge (Indiana University School of Medicine, Indianapolis, IN, USA). PA-1 cells were derived from a human teratocarcinoma, human ovarian cancer cells (Hey) were from a peritoneal deposit of a cytoadenocarcinoma of the ovary (from G Mills, MD Anderson Cancer Center, Houston, TX, USA), and a normal ovarian epithelial cell (IOSE80) was obtained from JA Hurteau (Department of Obstetrics and Gynecology, University of Illinois at Chicago, Chicago, IL, USA). All germline and ovarian cells were maintained in MEM supplemented with 10% fetal bovine serum at 37°C in a CO_2_ incubator, while IOSE 80 was maintained in MEM and 199/MCDB 105 (1 : 1) supplemented with 10% fetal bovine serum and EGF (10 ng ml^−1^).

*Germ cell tumours (GCTs)*: Tissue sections of biopsy materials with disseminated GCTs were obtained from the Indiana University Medical Center, University Hospital, under an Indiana University Institutional Review Board approved protocol (IU Study No. 9908-47) as 4% buffered formaldehyde-fixed tissues embedded in paraffin blocks, which were sectioned at 3 mm and fixed onto slides. Diagnosis was made from morphological examination of H&E-stained sections of biopsy material.

*Proteins, plasmids, chemicals, and antibodies*: Glutathione-*S*-transferase (GST) fusion form of c-Jun protein containing residues 1–79 of human c-Jun was overexpressed from *Escherichia coli* and purified using glutathione-agarose affinity column chromatography as described previously (Park *et al*, 2001). [*γ*-^32^P]ATP (4500 Ci mmol^−1^) was obtained from ICN. Adriamycin, EGF mitomycin C (MMC), and cisplatin were purchased from Sigma Chemical Co. (St Louis, MO, USA). Antibodies to EGFR, proliferating cell nuclear antigen (PCNA), c-Abl, Ku70/80, the catalytic subunit of DNA-dependent protein kinase (DNA-PKcs), JNK1 and/or JNK1/2 were obtained from either Santa Cruz Biotechnology (Santa Cruz, CA, USA) or Pharmingen (San Diego, CA, USA).

*Cell survival assay*: To examine drug resistance of cells, cells (1.0 × 10^4^ cells well^−1^) were plated in a 96-well plate and incubated for 24 h. Cells were treated with drugs and further incubated at 37°C and 5% CO_2_ for 72 h. After 72 h incubation, cell survival was measured using a colorimetric cell survival assay from Boehringer Mannheim (MTT Cell Proliferation Kit). Alternatively, clonogenic assay was used to measure the ability of cells to form colonies on 100 mm^2^ tissue culture dishes following treatment with ionising radiation or cisplatin. Controls consisted of cells untreated with peptides or DNA-damaging agent, or with neither. Cells were continuously exposed for 5 days to the indicated concentrations of the peptide, and colonies were stained with crystal violet and then colonies greater than 50 cells were counted. Each point represents mean values ±s.e., each conducted with triplicate plates.

*Immunohistochemistry*: Tissue sections were visualised for EGFR expression using an anti-EGFR monoclonal antibody (Santa Cruz Biotech., Santa Cruz, CA, USA). The Dako Universal Staining system (Dako Corp., Carpinteria, CA, USA) was used to automate the immunostaining procedure (Robertson *et al*, 2001). Sections were treated with 3% H_2_O_2_ for 10 min and incubated with an anti-EGFR antibody (1 : 1000) for 25 min, the biotinylatd goat anti-mouse antibody IgG secondary antibody for 10 min, streptavidin–horseradish peroxidase for 10 min, and diaminobenzidine for 5 min, according to Dako recommendation and empiric determination.

*JNK immunocomplex assay*: For JNK assay, cells were grown in culture media containing 0.5% fetal bovine serum for 16 h prior to the treatment with EGF or genotoxic agents. Cells were washed in ice-cold phosphate-buffered saline (PBS) and 0.5 ml of JNK lysis buffer (25 mM HEPES, pH 7.5, 0.3 M NaCl, 1.5 mM MgCl_2_, 0.2 mM EDTA, 0.5 mM dithiothreitol (DTT), 0.5% Triton X-100, 20 mM *β*-glycerophosphate, 1 mM sodium vanadate, 0.1 *μ*M okadaic acid, 1 mM phenylmethylsulphonyl fluoride, 20 *μ*g ml^−1^ aprotinin, 50 *μ*g/ml^−1^ leupeptin, and 10 *μ*M pepstatin) added to the dishes (150 × 25 mm) before scraping. After 30 min incubation on ice, insoluble materials were removed by centrifugation for 30 min at 12 000 r.p.m. JNK activity was determined by an immunocomplex assay essentially as described (Litz-Jackson *et al*, 1992; Duyster *et al*, 1995). Briefly, cell extracts (200 *μ*g) were mixed with 1.5 *μ*l of anti-JNK1/JNK2 polyclonal antibody for 1 h, and then 15 *μ*l of protein A–Sepharose beads was added and further incubated for 3 h at 4°C. The immunocomplex was washed three times with JNK lysis buffer and once with JNK kinase reaction buffer (20 mM HEPES pH 7.5, 10 mM MgCl_2_, 7 mM MnCl_2_, 1 mM EGTA, 1 mM sodium fluoride, 1 mM sodium vanadate, and 1 mM DTT). The precipitate was then resuspended in 30 *μ*l of JNK reaction buffer containing 2 *μ*g of GST-c-Jun (Park *et al*, 2001) and 50 *μ*M ATP and the reaction was initiated by the addition of 1.0 *μ*l of [*γ*-^32^P]ATP (45 000 Ci mmol^−1^). After incubation for 20 min at 30°C, the reaction was terminated by the addition of 8 *μ*l of 4 × SDS sample buffer (Laemmli, 1970) and heating to 95°C for 5 min. Samples were analysed on a 12% SDS–PAGE.

*Western blot analysis*: Extracts (40 *μ*g) from various ovarian cancer cells were loaded onto a 6 or 10% SDS–PAGE, and following gel electrophoresis proteins were transferred to nitrocellulose membrane and immunoblotted with primary antibody followed by a peroxidase-coupled secondary antibody (Amersham) and an enhanced chemiluminescence (Amersham) reaction prior to visualisation on a Kodak-o-mat film.

*Transfection and selection of stable cell lines*: Cells were transfected with either pEGFR-GFP or pEGFP-N3 using Lipofect AMIINE method (Life Technologies Inc.). Following antibiotic selection with G418 (600 *μ*g ml^−1^, Geneticin-Life Technologies, Gaithersburg, MD, USA), several EGFR-expressing clones were isolated and expanded into cell lines. Individual clonal lines expressing EGFR-GFP were established by plating a single cell into 96-well dishes. Cell clones expressing EGFR-GFP were utilised for the drug resistance study.

*Immunofluorescence microscopy*: PA-1 cells were grown on cover slides, washed twice with PBS, fixed in −10°C methanol for 5 min, air dried, and washed three times again with PBS. Fixed cells were incubated with an anti-EGFR polyclonal antibody (Santa Cruz Biotech.; 2 *μ*g ml^−1^) at room temperature for 1 h. After washing with PBS three times, cells were incubated for 1 h in the dark at room temperature with a 1 : 10 dilution of the secondary antibody (fluorescein-conjugated goat anti-mouse antibody; Oncogene Science) in PBS with 3% (w v^−1^) milk. Following extensive washing with PBS (five times), slides were prepared using 90% glycerol in PBS and stored in the dark at 4°C. Images were collected using a CCD 4910 camera with NIH image on a Zeiss Axiophot microscope.

*In vitro NER activity*: Reaction mixtures (50 *μ*l) contained 0.2 *μ*g each of UV-irradiated (450 J m^−2^) pBS (3 kb) and nonirradiated pBS (4.5 kb), 40 mM creatine phosphate-di-Tris salt (pH 7.7), 1 *μ*g creatine kinase, 50 mM HEPES-KOH (pH 7.8), 70 mM KCl, 7.5 mM MgCl_2_, 0.5 mM DTT, 0.4 mM EDTA, 2 mM ATP, 20 *μ*M of dGTP, dCTP, dTTP, 8 *μ*M of [*α*-^32^P dATP (25 000 cpm pmol^−1^), 5 *μ*g of BSA, and increasing amount of cell extracts (150 and 300 *μ*g) from various cells (Stigger *et al*, 1998). After incubation for 3 h at 30°C, DNA was isolated from the reaction mixtures, linearized with *Bam*H1, and separated on a 1% agarose gel electrophoresis in the presence of 0.5 *μ*g ml^−1^ ethidium bromide. Repair products were analysed by both fluorography and exposure to X-ray film.

## RESULTS

### Germline cells exhibit hypersensitivity to DNA-damaging drugs

**Figure 1:**
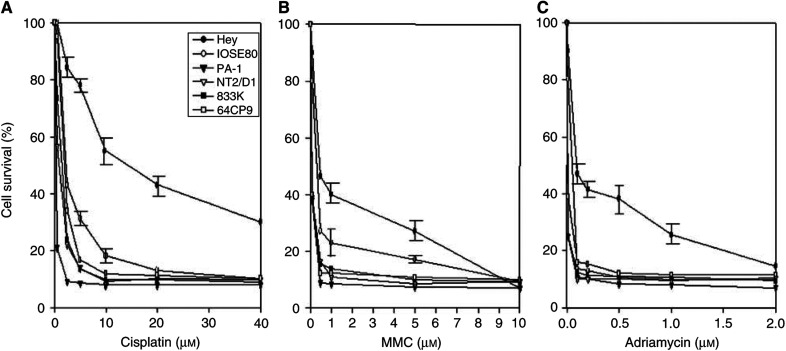
Effect of various drugs on the survival of GCTs (PA-1, 833K, NT2/D1, and 64CP9), ovarian primary epithelial cells (IOSE 80), and ovarian cancer cells (Hey). Cells were treated for 72 h with various concentrations of cisplatin (**A**), MMC (**B**), and adriamycin (**C**). Percentage of surviving cells was monitored using MTT assay and the results are the averages of three independent assays.

To analyse drug resistance of germline cells, four established germline tumour cells (PA-1, NT2/D1, 833K, and 64CP9) were compared with a normal ovarian epithelial cell line (IOSE80) and drug-resistant ovarian cancer cells (Hey) derived from a peritoneal deposit of a cytoadenocarcinoma of the ovary (Figure 1). The established ovarian cancer cells (Hey) showed a marked resistance to cisplatin treatment, while the germline tumour cells were remarkably sensitive to the drug treatment (Figure 1). All four germline tumour cells showed extreme sensitivity to cisplatin treatment (5 *μ*M) with a survival rate of less than 10%, whereas 80% of Hey cells survived under the same conditions. Meanwhile, a primary epithelial ovarian cell (IOSE80) showed a medium level of cell survival following cisplatin treatment (Figure 1). Adriamycin is a DNA-intercalating agent that causes DNA strand break damage, while MMC mainly causes DNA damage by forming a DNA crosslink. Similar to the cisplatin treatment, germline tumour cells were highly sensitive to both MMC and adriamycin treatment (Figure 1B and C).

### Drug sensitivity of germline cells correlates with the lack of EGFR expression

**Figure 2:**
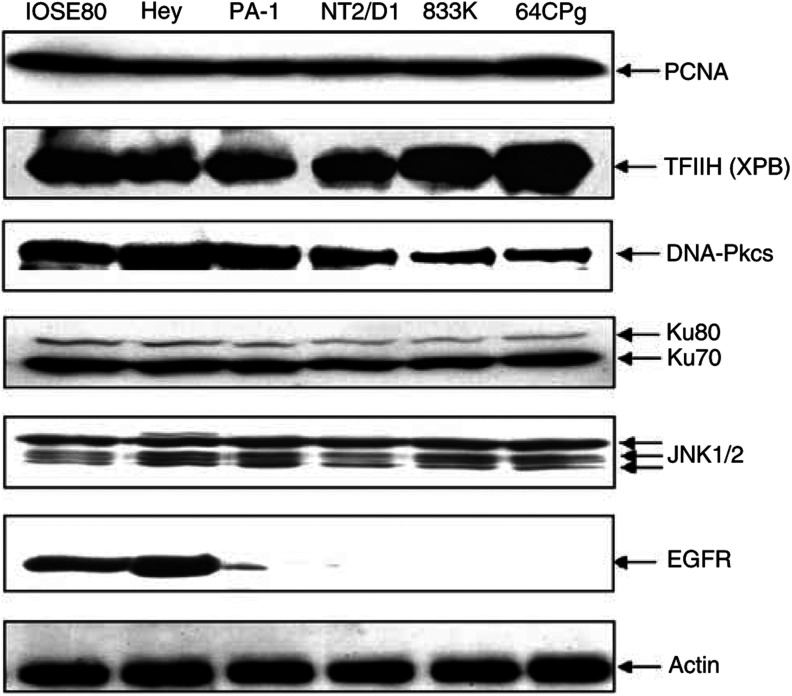
Expression of various proteins in germline cells. Extracts (100 *μ*g) from various germline tumour cells (PA-1, 833K.NT2/D1, and 64CP9) and ovarian cells (Hey and IOSE-80) were analysed for the expression of DNA repair factors or damage signalling proteins by Western blot.

To better understand the hypersensitivity of germline tumour cells to DNA-damaging drug, we analysed expression of various proteins that are involved in the drug sensitivity of cells. No noticeable difference was observed between drug-sensitive germline tumour cells and a drug-resistant cell (Hey) in the expression of DNA repair proteins (PCNA, TFIIH, DNA-PKcs, and Ku70/80) (Figure 2). We noticed however some difference in the expression of DNA-PKcs (Figure 2), although this subtle difference was not consistently observed in multiple experiments (data not shown). Also, we did not see any significant difference between germline cells and Hey cells in the *in vitro* NER activity (data not shown). Interestingly, a significant difference was observed in the expression of EGFR between germline tumour cells and ovarian cancer (Hey) cells, while the expression of JNK1 and JNK2 showed no difference between them (Figure 2).

### Expression of EGFR enhances the drug resistance of germline cells

**Figure 3:**
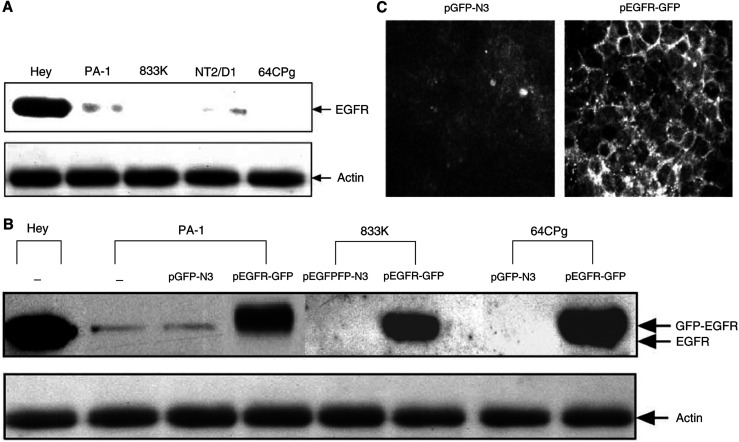
Whole-cell lysates (30 *μ*g) from various germline tumour cells (PA-1, 833K, NT2/D1, and 64CP9) and Hey cells were examined for the expression of EGFR by Western blot (**A**). In (**B**), PA-1 cell lines were stably transfected with either pEGFP-N3 vector or pEGFR-GFP, while 833k and 64CP9 cells were transiently transfected for 36 h with pEGFP-N3 or pEGFR-GFP (see ‘Materials and Methods’ for the details). Expression of EGFR-GFP was monitored by Western blot. (**C**) shows the expression of GFP (left) or GFP-EGFR (right) in PA-1 cells that were stably transfected with pEGFP-N3 or pEGFR-GFP, respectively. For immunofluorescence, cells were fixed and permeabilised briefly with methanol incubated with anti-EGFR antibody as described under ‘Materials and Methods’.

To further examine whether the lack (or low level) of EGFR expression in germline tumour cells (Figure 3A) contributes to their drug sensitivity, cells were transfected with plasmid DNA expressing either green fluorescence protein (GFP) or GFP-EGFR fusion protein and analysed for their effect on drug resistance of cells. After initial selection of cells expressing GFP or GFP-EGFR, protein expression and cellular localization were analysed by Western blot (Figure 3B) and by fluorescence microscopy (Figure 3C), respectively. Germline tumour cells harbouring pEGFR-GFP plasmid showed a high level of EGFR expression, which was comparable to that in drug-resistant ovarian cancer (Hey) cells (Figure 3B). Cells harbouring pEGFR-GFP not pEGFP-N3 showed EGF-dependent activation of JNK1, suggesting that GFP-EGFR fusion protein is functionally active (data not shown).

**Figure 4:**
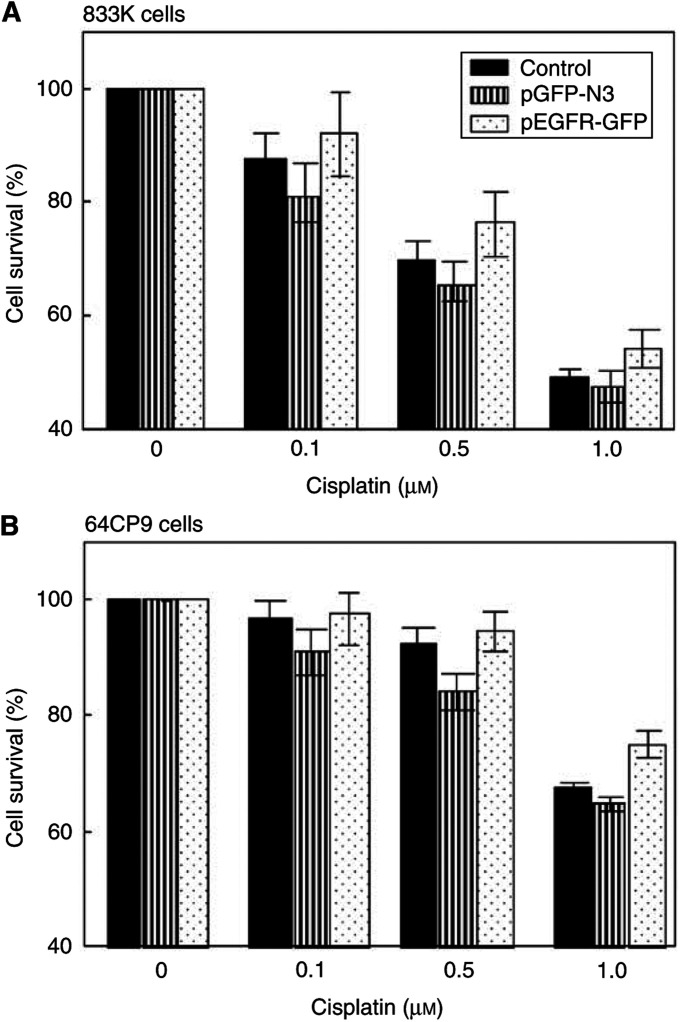
Transient expression of EGFR enhances the survival of two germline tumour cells (833K (**A**); 64CP9 (**B**)) following cisplatin treatment. Control cells were compared with those transiently transfected with either pEGFP-N3 vector or pEGFR-GFP and examined for their cell survival following cisplatin treatment. At 24 h after transfection, cells were exposed to the indicated amount of cisplatin for 72 h. The percentage of surviving cells was monitored by MTT assay.

**Figure 5:**
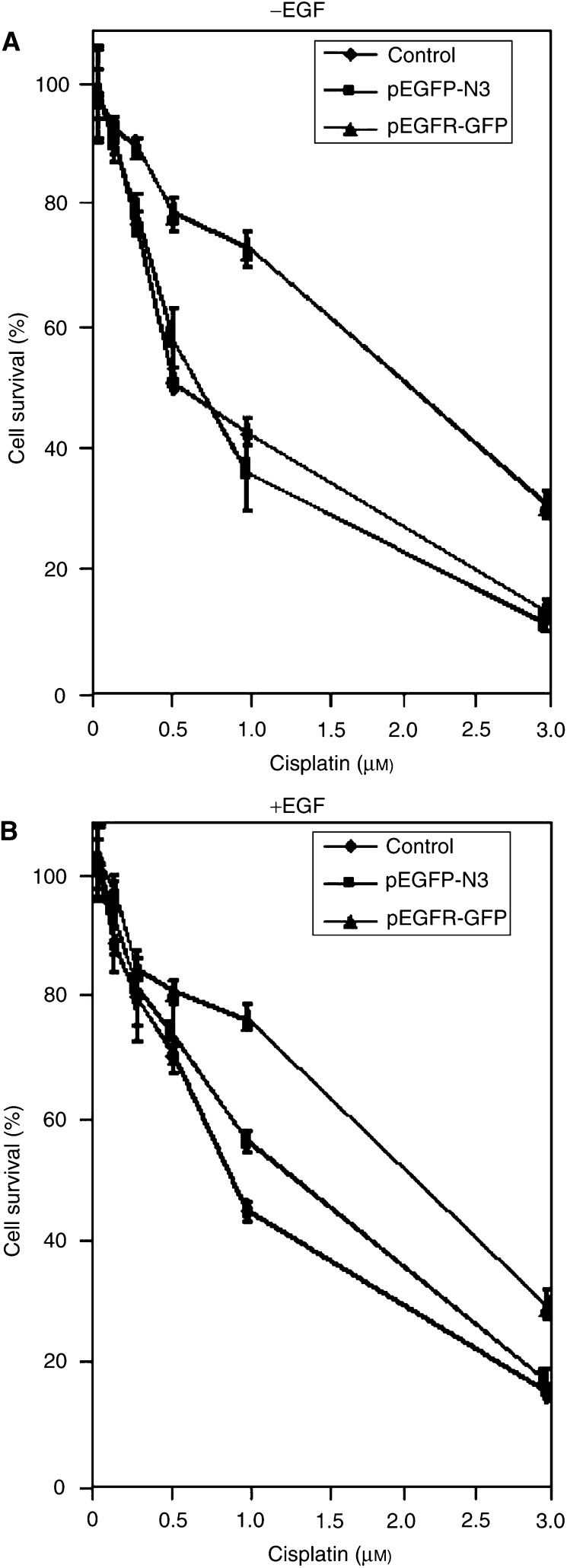
Overexpression of EGFR markedly increased the survival of PA-1 cells following cisplatin treatment. Control cells (PA-1) were compared with those stably transfected with either pEGFP-N3 vector or pEGFR-GFP for their cell survival in the presence (**A**) and absence (**B**) of EGF following cisplatin treatment. At 24 h after the seeding, cells were exposed to the indicated amount of cisplatin for 7 days before counting colonies (>50 cells colony^−1^). Each point is the mean value of triplicate experiments.

Germline cells transfected with pEGFR-GFP showed only a marginal increase in their cell survival following cisplatin treatment, while cells expressing GFP (pEGFP-N3) exhibited a slight decline in cell survival (Figure 4 and Table 1). When a stably transfected cell instead of transient system was examined for drug sensitivity, however, it not only showed a significant increase in EGFR expression, but also enhanced survival of germline tumour cells following cisplatin treatment (Figure 5 and Table 1). Although EGFR kinase is activated by EGF, we did not see a substantial increase in cell survival in the presence of EGF probably because EGFR can also be activated by cisplatin. The difference in cell survival between transiently transfected cells (Figure 4) *vs* stable transfectants (Figure 5) following drug treatment may be due to the lower transfection efficiency in transient system, where only 30% of cells expressed GFP-EGFR (data not shown). Together, our results suggest that (1) a lack (or lower level) of EGFR expression in germline tumour cells contributes to their drug sensitivity and (2) EGFR may play a positive role in protecting cells following treatment of cells with DNA-damaging agent.

### Lack (or lower level) of EGFR expression in primary germline cells

To see whether lack or lower level of EGFR expression is a common property of germline tumour cells, a number of primary GCTs were selected and tested for EGFR expression. Among 61 GCTs tested, 35 showed undetectable level of EGFR expression, while the remaining samples expressed very low level of EGFR compared to a control ovarian cancer cells (Table 2), supporting the observation with established cells (PA-1, NT2/D1, 833K, and 64CP9) that germline tumours express lower level of EGFR (Figure 3). In fact, the probability of all 61 GCT samples having EGFR expression no higher than+is extremely low (2 × 10^−25^).

## DISCUSSION

Alteration of DNA repair factors or damage response proteins has been associated with drug resistance of cancer cells (Mohrenweiser *et al*, 2003). For example, a tumour suppressor gene, p53, is a key DNA damage mediator that plays a dual role following exposure to cytotoxic treatment (Ferrera *et al*, 1999); it is involved in damage-induced apoptosis, but also plays a role in cell cycle arrest and DNA repair, cellular processes that can affect the sensitivity to chemotherapeutic drug. However, a consensus on the role for DNA repair genes in drug resistance of various cancer cells has not been reached, mainly because the complicated nature of drug-induced resistance with various tumours made it difficult to delineate a single mechanism (such as DNA repair) that contributes to the resistance.

Compared to drug-resistant ovarian cancer cells, germline tumour cells showed a marked sensitivity following the treatment with cisplatin, adriamycin, or MMC (Figure 1). Examination of the established cell lines as well as primary germcell tumours for genetic alteration of several key repair factors and damage signalling factors indicated that drug sensitivity of germline tumour cells may not be due to an alteration of repair factors or DNA repair activity (Figure 2). Instead, there was a good correlation between EGFR expression (or EGF-induced JNK activation) and drug resistance among ovarian and germline tumour cells. Low level of EGFR expression in germline tumour cells may be linked to their drug sensitivity and supports a positive role for EGFR in drug resistance of cancer. The latter may be explained by the fact that EGF and its receptor activate the JNK signalling pathway that leads to the induction of genes involved in DNA repair and cellular redox (Adler *et al*, 1992; Foltz *et al*, 1998; Roulston *et al*, 1998).

Epidermal growth factor receptor is a 170 kDa transmembrane glycoprotein with tyrosine kinase activity. Although EGFR was shown to have no independent prognostic significance in advanced cancer (Baekelandt *et al*, 1999), the EGFR and HER2/neu were frequently overexpressed in malignant tumours. Recent microassay analysis revealed that amplification of EGFR gene was found in many tumours including ovarian cancer (Lei *et al*, 1999), glioblastoma (Hui *et al*, 2001), pancreatic cancer (Bruell *et al*, 2003; Schreiner *et al*, 2003), gastric cancer (Garcia *et al*, 2003), prostate cancer (Skacel *et al*., 2001), and lung adenocarcinoma and head/neck squamous cell carcinoma (Haedicke *et al*, 2003; Shintani *et al*, 2003), suggesting that overexpression of EGFR may be linked to the oncogenesis of various cancers. High level of EGFR expression also correlates with increased tumour resistance to radiation (Shintani *et al*, 2003), suggesting that EGFR may mediate radioresistance of cancer cells (Liang *et al*, 2003). Epidermal growth factor receptor is also a cellular receptor for human cytomegalovirus, a cancer-causing virus that causes severe and fatal disease in immune-comprimised individuals (Wang *et al*, 2003).

Epidermal growth factor receptor-associated protein tyrosine kinase complexes also have vital antiapoptotic functions in human breast cancers (Modjtahedi *et al*, 1998; Witters *et al*, 1999) and the blockade of EGFR not only adversely affected cell growth, but also showed a sign of terminal differentiation and induces apoptosis in the human cancer cells (Modjtahedi *et al*, 1998). Similarly, drug-induced apoptosis in human breast cancer cells was abrogated by using EGFR antisense RNA (Dixit *et al*, 1997), suggesting that a critical level of EGFR signalling, which is amplified in some common cancers, may be necessary for DNA-damaging drug-mediated apoptosis in tumour cells and suggest an inhibitory effect of this pathway on the repair of cisplatin-damaged DNA. In fact, cancer cells expressing higher levels of EGFR were much more resistant to the growth inhibitory effect of DNA-damaging agents than were control cells (Dixit *et al*, 1997).

Various strategies have been developed to target EGFR and to deter cancer cell growth (Zhang *et al*, 2000; Bruell *et al*, 2003; Heimberger *et al*, 2003). For example, the treatment of cancer cells with EGFR tyrosine kinase inhibitor markedly potentiates the efficacy of many cytotoxic agents against several human cancer xenografts (She *et al*, 2003). The use of antisense oligonucleotides or monoclonal antibodies to EGFR also showed significant inhibition of cancer cell growth (Modjtahedi *et al*, 1998; Witters *et al*, 1999), while activation of EGFR family members suppresses the cytotoxic effects of TNF-alpha (Hoffmann *et al*, 1998).

Although mutations in proto-oncogenes (c-*ret*) as well as DNA MMR genes have been linked to germline tumours (van Puijenbroek *et al*, 1997; Leung *et al*, 2000), alteration of EGFR in germcell tumours has not been reported. This study showed that germline tumour cells not only exhibited lower EGFR expression but also were highly sensitive to DNA-damaging drugs, suggesting that the lack of EGFR expression contributes at least in part to the drug sensitivity of germline cells.

## Figures and Tables

**Table 1:** Effect of EGFR expression on cisplatin resistance of germline tumour cells (833 K) following cisplatin treatment

**Cisplatin (*μ*M)**	**Mean cell survival rate (%)**		
**(−EGF)**	**pEGFP-N3**	**pEGFP-GFP**	***P*-value from *t*-test**
0.1	81.25	95.5	0.006
0.5	69.25	78	0.008
1.0	49.25	57.25	0.002
			
(+EGF)			
0.1	93.25	98	0.047
0.5	87.75	97.5	0.001
1.0	64.25	75.25	0.004

Cells expressing EGFR (pEGFR-GFP) were compared with control cells (pEGFP-N3) for cell survival following cisplatin treatment in the presence and absence of EGF (*n*=4).

**Table 2:** Germ cell tumour (GCT) samples scored for EGFR expression by immunohistochemistry

**GCT#**	**EGFR**	**GCT#**	**EGFR**	**GCT#**	**EGFR**
LSAB	++++	GCT 1218	+	GCT 1191	−
GC control	+	GCT 1108-2	+	GCT 1171	−
GCT 1221	+	GCT 1131-2	−	GCT 1169	−
GCT 1130-2	+	GCT 1132	−	GCT 1190	−
GCT 1115	−	GCT 1142	+	GCT 1183	−
GCT 1230-2	+	GCT 1116	−	GCT 1182	−
GCT 1206	+	GCT 1124	−	GCT 1181	+
GCT 1110	+	GCT 1127	−	GCT 1156	+
GCT 1208	+	GCT 1118	−	GCT 1122	+
GCT 1106	−	GCT 1229	−	GCT 1199	−
GCT 1108	+	GCT 1144	−	GCT 1198	−
GCT 1135	−	GCT 1202	+	GCT 1160	+
GCT 1143-2	−	GCT 1154	−	GCT 1204	−
GCT 1134	−	GCT 1153	−	GCT 1224	+
GCT 1215	−	GCT 1151	−	GCT 1158	+
GCT 1182-2	+	GCT 1150	−		
GCT 1212	+	GCT 1160-2	−		
GCT 1145-2	+	GCT 1147	+		
GCT 1125	+	GCT 1161-2	−		
GCT 1097-2	+	GCT 2470-9	−		
GCT 1098	+	GCT 1165	−		
GCT 1100	+	GCT 1166	−		
GCT 1138	−	GCT 1168	−		
GCT 1214	−	GCT 1194	−		

Two control cells (ovarian cancer cells (Hey, LSAB) and a normal germ cell, GC control) were included to compare the level of EGFR expression in GCT. ‘−’ represents no detectable EGFR expression, while the level of protein expression was determined by comparing with two control cells, GC control at the lowest level (+) and LSAB at the highest level (++++).
